# Histological Findings and T2 Relaxation Time in Canine Menisci of Elderly Dogs—An Ex Vivo Study in Stifle Joints

**DOI:** 10.3390/vetsci10030182

**Published:** 2023-02-24

**Authors:** Lena Bunzendahl, Amir Moussavi, Martina Bleyer, Jana Dehnert, Susann Boretius, Stephan Neumann

**Affiliations:** 1 Small Animal Clinic, Institute of Veterinary Medicine, Georg-August University of Göttingen, 37077 Göttingen, Germany; 2 Functional Imaging Laboratory, German Primate Center, Leibniz Institute for Primate Research, 37077 Göttingen, Germany; 3 Pathology Unit, German Primate Center, Leibniz Institute for Primate Research, 37077 Göttingen, Germany

**Keywords:** osteoarthritis, canine, menisci, stifle, magnetic resonance imaging, functional imaging, T2, histological score, meniscal degeneration

## Abstract

**Simple Summary:**

Osteoarthritis is a common disease in dogs, most often affecting the stifle joint and causing damage to all joint structures. Detecting early stages of osteoarthritis is important for an effective treatment. Conventional magnetic resonance imaging (MRI) is the golden standard imaging technique for diagnosing pathologies of soft tissues in the stifle joint. However, it is limited to the visualization of macroscopic tissue pathology. In contrast, quantitative MRI offers a more sensitive method for diagnosing early pathological changes, as it enables the detection of microstructural processes. The menisci play an important role in joint health. They undergo structural changes in osteoarthritis, including alterations in water content and collagen structures, as well as deviations in proteoglycan content. Different studies have shown the potential of special MRI sequences to identify these changes, e.g., T2 relaxation time. In this study, canine menisci of elderly dogs without clinical evidence of hindlimb lameness were examined histologically and using MRI. Our results showed that clinically healthy elderly dogs exhibited slight histological, probably age-related, degenerative changes in the menisci, but did not reveal significant radiological evidence of chronic inflammatory and degenerative changes, including no significant changes in T2 relaxation time.

**Abstract:**

Osteoarthritis is a chronic disease that often affects the canine stifle joint. Due to their biomechanical function, the menisci in the canine stifle play an important role in osteoarthritis. They compensate for the incongruence in the joint and distribute and minimize compressive loads, protecting the hyaline articular cartilage from damage. Meniscal degeneration favors the development and progression of stifle joint osteoarthritis. Qualitative magnetic resonance imaging (MRI) is the current golden standard for detecting meniscal changes, but it has limitations in detecting early signs of meniscal degeneration. A quantitative MRI offers new options for detecting early structural changes. T2 mapping can especially visualize structural changes such as altered collagen structures and water content, as well as deviations in proteoglycan content. This study evaluated T2 mapping and performed a histological scoring of menisci in elderly dogs that had no or only low radiographic osteoarthritis grades. A total of 16 stifles from 8 older dogs of different sex and breed underwent ex vivo magnet resonance imaging, including a T2 mapping pulse sequence with multiple echoes. A histological analysis of corresponding menisci was performed using a modified scoring system. The mean T2 relaxation time was 18.2 ms and the mean histological score was 4.25. Descriptive statistics did not reveal a correlation between T2 relaxation time and histological score. Ex vivo T2 mapping of canine menisci did not demonstrate histological changes, suggesting that early meniscal degeneration can be present in the absence of radiological signs of osteoarthritis, including no significant changes in T2 relaxation time.

## 1. Introduction

Osteoarthritis is a chronic progressive disease that leads to changes in all joint compartments. It is currently incurable and causes irreversible damage to the affected joint. In particular, the stifle joint, as a complex synovial joint, is frequently affected in both humans and dogs. In dogs, osteoarthritis of the stifle joint mainly occurs in cases of ruptured cranial cruciate ligament and patellar luxation [1]. Due to its chronic and progressive character, osteoarthritis causes severe pain and significant restrictions on quality of life.

The menisci in stifle osteoarthritis have two pathogenetic roles. These meniscal tears can both be a consequence of osteoarthritis and can also contribute to its development [2,3,4]. Therefore, the menisci are the object of this current research, which focuses on the pathogenesis and mechanisms of osteoarthritis.

The menisci of the stifle joint are tangerine-shaped fibrocartilage discs located on the medial and lateral sides of the tibial plateau. They have important functions in the biomechanics of the stifle joint. They compensate for the incongruence in the joint and distribute and minimize compressive loads so that the hyaline articular cartilage is protected from damage [5,6]. Therefore, the menisci enable physiological motion in the stifle joint and protect the joint from impact and compressive loads. Intact menisci are therefore a fundamental requirement for the long-term maintenance of stifle joint health [7]. In the context of osteoarthritis, morphological, biomechanical and molecular changes occur in all joint compartments, including the menisci. An objective assessment of the structure and composition of the meniscal tissue can be an important prognostic marker with regard to the detection of early changes in the joint. Particularly in patients at risk of osteoarthritis, early detection and therapeutic intervention decelerate disease progression [7,8]. Magnetic resonance imaging (MRI) has become the diagnostic modality of choice for detecting intra-articular abnormalities in the stifle joint [9]. Conventional qualitative MRI scans are limited to the detection of degeneration that results in macroscopic morphological changes [10,11,12]. These include, for example, tears of the meniscus or signs of degeneration that lead to a loss of substance in the entire meniscus. In contrast, quantitative MRI represents a different method in this research field, as it detects structural changes in the biochemical composition of affected organs [11]. Changes in the menisci that occur in the course of degenerative joint changes primarily take place in the extracellular matrix and consist of altered water contents, altered collagen structures and deviations in proteoglycan contents. These changes can be detected, for example, using T2 relaxation time [10,13]. Recent studies have shown that T2 relaxation time has the potential to serve as a biomarker for early degeneration of meniscal tissue [10,13,14,15,16]. Previous studies have focused on human menisci. To our knowledge, validation of ex vivo T2 mapping in canine menisci has not been performed yet. This study evaluated histological degenerations in menisci of elderly lame dogs that had no or mild radiological signs of osteoarthritis. A radiological evaluation was performed using conventional X-rays and MRIs, including T2 mapping of the medial and lateral menisci.

## 2. Materials and Methods

### 2.1. Study Population

For this study, 16 stifle joints of 8 older dogs (aged between 10 and 17 years) of both sexes from different breeds were examined. The body weight varied from 15 to 30 kg and body condition scores (BCS) ranged from 4 to 7. We included 1 “Labrador Retriever”, 1 “Deutsche Wachtel”, 1 “Bayerischer Gebirgsschweißhund”, 1 “Boxer” and 4 “mixed-breed dogs”. We only examined stifle joints from dogs that were euthanized at the small animal clinic of the Institute of Veterinary Medicine, Georg-August University of Göttingen, from 2019 to 2022, for reasons unrelated to this study. There was no evidence of existing hindlimb lameness in their medical history and no signs of instability in their stifle joints.

### 2.2. Sample Preparation

After they were euthanized, the dogs were stored in a cold room at 8 °C. The stifle joints were dissected from surrounding soft tissue no later than 24 h post mortem and placed in a container filled with 10% neutral-buffered formaldehyde. In addition, depending on the size of the joint, 1–2 mL of the formaldehyde solution was injected into the joint to fix intraarticular structures as efficiently as possible. During the intra-articular injection, strict care was taken not to introduce any air into the joint.

### 2.3. X-ray Grading

**Figure 1:**
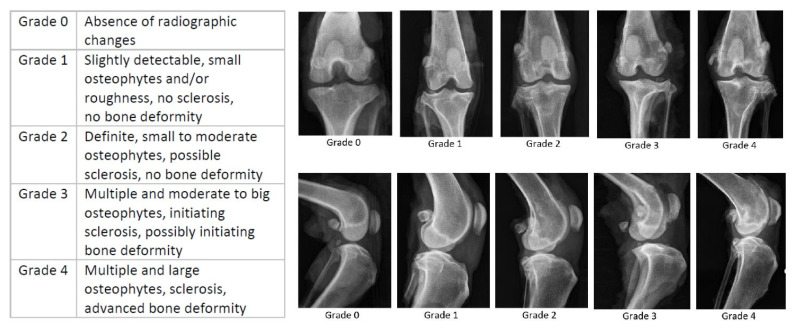
Modified radiographic osteoarthritis grading system for dogs [17] according to the Kellgren–Lawrence system [18], and exemplary pictures for each osteoarthritis grade. In this study, only dogs with grade 0 and grade 1 were included.

The dissected joints were radiographed in medio-lateral and cranio-caudal beam paths. A radiographic assessment of the degree of osteoarthritis was performed using a modified Kellgren–Lawrence grading system [17]. The definition of an osteoarthritis grade and its exemplary pictures are illustrated in Figure 1. Only dogs with no or mild radiographic evidence of osteoarthritis were included in the study.

### 2.4. MR Imaging

Formalin-fixed joints were examined with a 3 T whole-body MRI (MAGNETOMPrisma, Siemens Healthineers, Erlangen, Germany) with a maximum gradient strength of 80 mT/m. Images were acquired with a 16-channel coil (Variety, NORAS MRI products GmbH, Höchberg, Germany) in which the container with the joints was fixed. The MR imaging protocol included T2-weighted sequences in a sagittal, coronal and axial plane. For slice positioning, low-resolution gradient echo images were acquired in three orientations (axial, sagittal and coronal). Afterward, sagittal 2D multi-echo, spin-echo images (TR= 7000 ms, TE = n × 13.5 ms, 12 echoes, spatial resolution = 0.32 × 0.32 mm², field of view = 144 × 144 mm², slice thickness f 0.9 mm, 48 slices, pixel bandwidth = 310 Hz/Px) were acquired and maps of T2 relaxation times were calculated using an in-house pixelwise mono-exponential fitting routine that ignored the first echo time (Matlab, R2021a, MathWorks, Natick, MA, USA).

For the medial and lateral meniscus, a region-of-interest (ROI) was manually drawn in each image dataset using the program “ITK-Snap”. The segmentation was based on the T2-weighted sequence, which provided the best possible contrast between the menisci and the surrounding tissue. There were two ROIs per joint, the medial and lateral meniscus.

### 2.5. Histology

**Figure 2:**
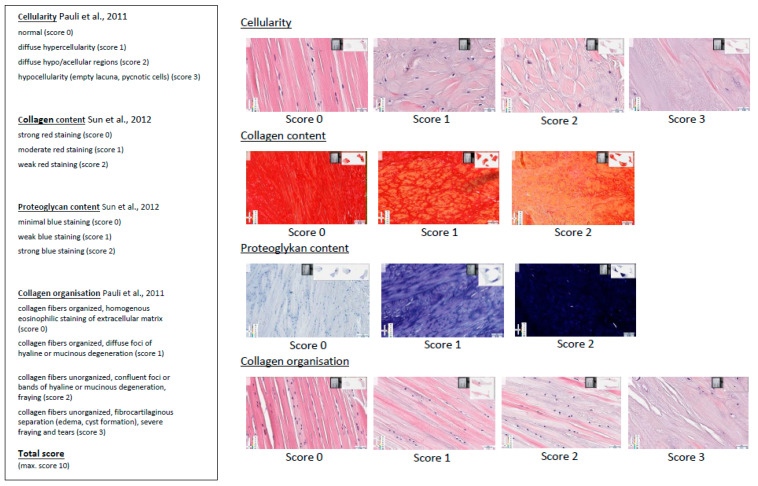
Modified histological scoring system according to [23,24].

Following the MRI measurements, menisci were prepared for histological examination. Medial and lateral menisci were harvested from each joint and sectioned longitudinally to allow assessment of the anterior and posterior horns as well as the middle section. The obtained samples were again fixed in 10% neutral-buffered formaldehyde, embedded in paraffin and sectioned at approximately 4 μm. A macroscopic examination of the cranial cruciate ligament was performed to verify that there was no evidence of a partial or complete tear. Sections were stained with hematoxylin–eosin (H&E) to provide an overview of the specimens and for the evaluation of cellularity and collagen organization. In addition, the qualitative assessment of collagen content was performed using picrosirius red staining [19,20,21], while the qualitative assessment of proteoglycan content was performed using toluidine blue staining. For the picrosirius red, a staining kit from SkyTek Laboratories was used (ScyTek Laboratories, Inc. 205 South 600 West, Logan, UT, USA). An aqueous toluidine blue stain was performed according to a standard protocol [22]. The visualization of stained sections was performed using digital microscopy. Samples were scanned by an Aperio CS2 eSlide Scanscope (Leica Biosystems, Nussloch, Germany) and viewed with Aperio eSlide Manager. The histological evaluation was based on a modified scoring adapted from [23,24] (Figure 2). One slice from each meniscus and stain was evaluated. After evaluating the menisci, a total score was created by adding the scores of each category. A maximum total score of 10 corresponded to the maximal degree of degeneration.

All samples were independently evaluated by two examiners with different levels of experience. In case of questionable results, the assessment was re-performed together so that a consensus could be reached.

### 2.6. Statistical Analysis

We performed a descriptive statistical analysis. Histological findings and T2 values were described for medial and lateral menisci in all patients.

## 3. Results

### 3.1. Study Population

In this study, a total of 32 menisci, 16 medial and 16 lateral, from 16 canine stifle joints of 8 dogs were examined. The joints were from dogs of different breeds, male and female. Detailed information about the dogs is shown in Table 1.

### 3.2. Radiographic Osteoarthritis Score

Overall, 12 out of 16 (75%) of the included joints had mild radiographic signs of osteoarthritis, corresponding to Kellgren–Lawrence grade 1. The four other joints (25%) had no evidence of osteoarthritis, classifying as grade 0.

### 3.3. T2 Relaxation Time in Meniscal Tissue

**Figure 3:**
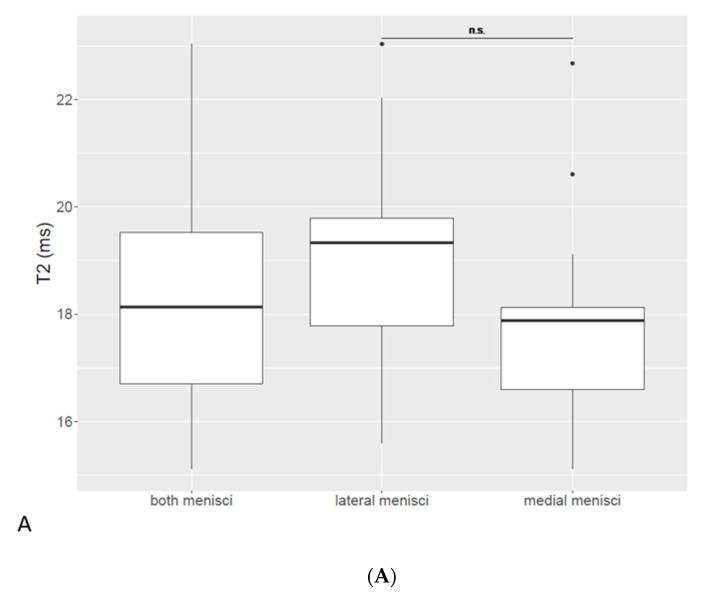

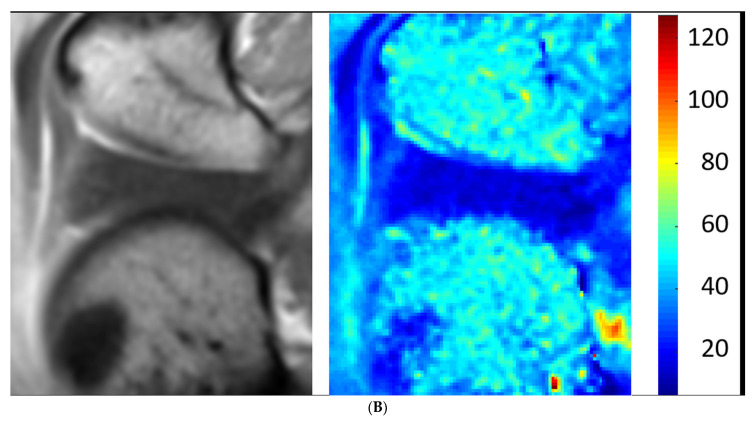
(**A**) Meniscal T2 values in both medial and lateral menisci. There was no significant difference between medial and lateral menisci (*p* = 0.13). (**B**) Sagittal magnitude image of a stifle joint and corresponding T2 map. Color chart on the right side represents T2 relaxation times in ms.

The mean T2 relaxation time was 18.3 ms (range 15.1–23). In medial menisci, the mean T2 relaxation time was 17.7 ms (range 15.1–22.7), while it was 19.0 ms (range 15.6–23) in lateral menisci. There was no significant correlation between T2 values and age, body weight or body condition score (BCS). Representative findings of T2 mapping are displayed in Figure 3. The paired t-test showed no significant difference between medial and lateral menisci (*p* = 0.13).

### 3.4. Histological Findings

**Figure 4:**
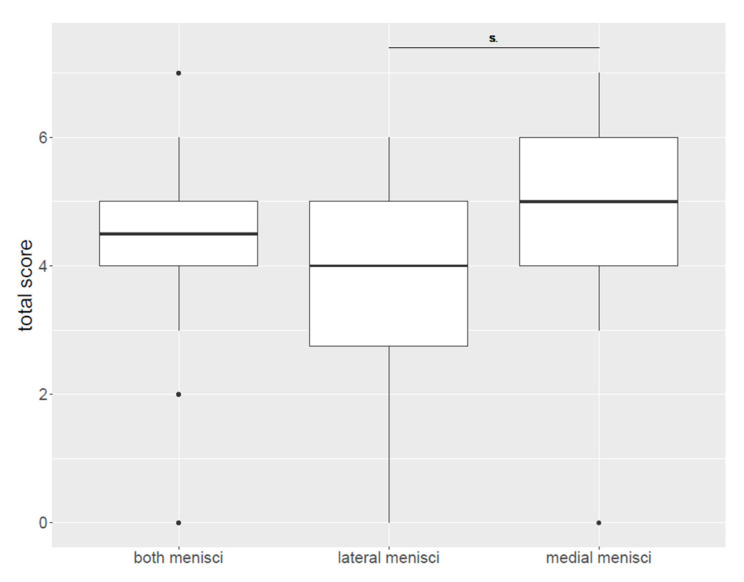
Total histological degeneration score in medial and lateral menisci. There was a significantly higher degeneration score in medial menisci than in lateral menisci (*p* = 0.006).

**Figure 5:**
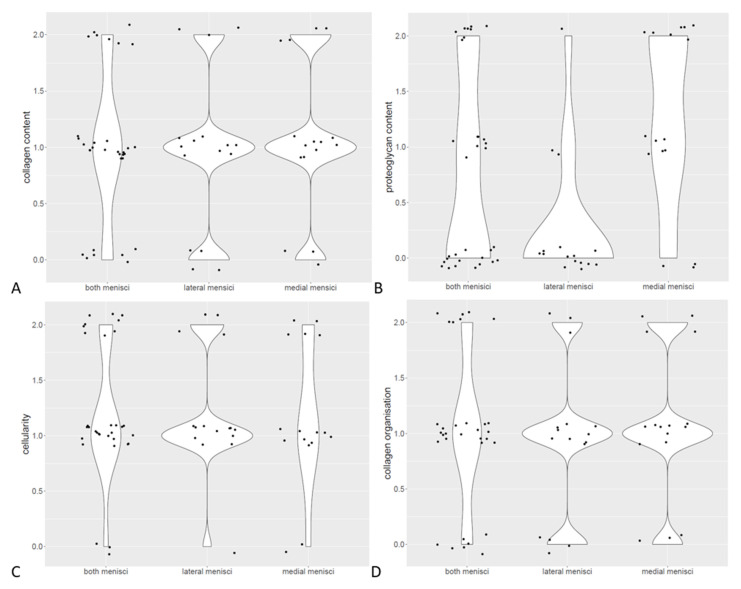
Scoring of collagen content showed no significant differences between medial and lateral menisci (**A**). Scoring of proteoglycan content showed a higher score in lateral menisci (**B**). Scoring of cellularity showed no significant differences between medial and lateral menisci (**C**). Scoring of collagen organization showed no significant differences between medial and lateral menisci (**D**).

All 32 menisci were examined histologically using the three different stains. An evaluation of degeneration showed an overall mean histological score of 4.25. Medial menisci had a mean score of 4.75 and lateral menisci had a mean score of 3.75. The histological score details for the total score are shown in Figure 4. The paired *t*-test showed a significant difference between medial and lateral menisci (*p* = 0.006). The content of collagen and proteoglycans varied from score 0 to 2. The collagen organization varied from score 0 to 3 and cellularity showed a degeneration score between 0 and 2. Detailed results of each histologcal score are presented in Table 2, Table 3 and Table 4 and Figure 5. There was no significant correlation between total histological score, collagen content, proteoglycan content, cellularity, collagen organization and age, weight or BCS.

### 3.5. T2 Relaxation Time and Histological Findings

**Figure 6:**
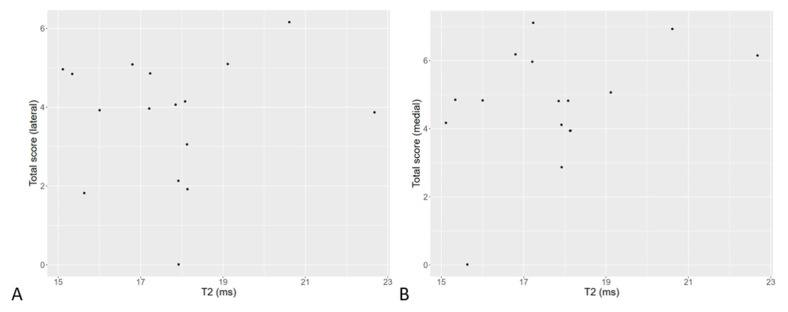
T2 relaxation time versus total histological score in lateral (**A**) and medial (**B**) menisci. There was no significant correlation between T2 values and histological scores.

There was no significant correlation between histological findings and T2 relaxation time in the examined menisci. The potential correlation was examined for T2 relaxation time and total score, collagen content, proteoglycan content, cellularity and collagen organization. Detailed results for the T2 values and total histological score are shown in Figure 6.

## 4. Discussion

This study was conducted to assess the presence and extent of histological degeneration of menisci in older lameless dogs. Only dogs with low-grade radiographic stifle osteoarthritis were included. Our results demonstrated that there were distinct histological signs of degeneration in the studied menisci, even if there were no obvious signs of osteoarthritis in imaging modalities. These findings suggest that there can be different degrees of degeneration in the menisci of older dogs before clinical signs of lameness or radiographic evidence of osteoarthritis develop. Meniscal degeneration has been studied in dogs before, but only a minority of these studies performed both histological and magnetic resonance imaging (MRI) [25,26,27]. Collagen fibers and proteoglycans are important components of the meniscal extracellular matrix. Changes in their composition can have a strong impact on the mechanical strength of the meniscus. For example, collagen content in osteoarthritic menisci decrease while the disorganization of collagen fibers increase. Additionally, the content of collagen decreases in osteoarthritic menisci, which causes a diminution of mechanical strength. In human menisci, a reduced content of collagen and proteoglycans has been described in the context of degeneration [13,23,28,29]. Others reported a decrease in collagen content, but an increase in proteoglycan content in osteoarthritic menisci [23]. There are some studies, however, that have examined quantitative MRI, such as T2 mapping, as a biomarker for meniscal or cartilage degeneration [1,30,31,32,33,34,35]. Histological grading has not been performed regularly, but most studies demonstrated that T2 mapping can differentiate between healthy patients and patients with osteoarthritis. Mittal et al. [35] demonstrated that T2 mapping reflects changes in the biochemical composition of menisci. The authors suggested that T2 values are sensitive to interactions between water molecules and the collagen network, but cannot reflect changes in proteoglycan content [35]. Zarins et al. [34] showed that T2 measurements can be a non-invasive technique to detect and quantify meniscal degeneration. Among other imaging parameters, Rauscher et al. [15] evaluated T2 mapping in menisci of patients with varying degrees of osteoarthritis and compared the results with those of a healthy control group. They found that meniscal T2 values correlated with clinical signs of osteoarthritis, thereby distinguishing between healthy patients and patients with mild to severe meniscal degeneration. They also concluded that T2 mapping might be more useful than other imaging parameters for differentiating between the investigated groups [15]. In addition to osteoarthritis patients, quantitative MRI measurements were carried out on patients with acute stifle injury. Wang et al. [14] reported a significant increase in meniscal T2 values in these patients. Even in patients without morphological changes, values were higher than those of the healthy control group [14].

Eijgenraam et al. [30] were the first to correlate meniscal T2 mapping with histological grading as the reference standard, while the imaging was performed in vivo. Nebelung et al. [16] performed a comprehensive validation study for multiple quantitative MRI parameters, using histological grading as the reference standard. In contrast to Eijgenraam et al. [30], they performed the imaging ex vivo. It must be considered that all the aforementioned studies were performed on human patients. The present study is, therefore, one of the first to examine canine menisci with quantitative MRI, using histological scoring as the reference standard. Comparisons with other studies must be viewed critically as there are differences between humans and dogs. Additionally, some studies were performed ex vivo and others in vivo. It is questionable as to whether or not the T2 measurements obtained ex vivo reflect the in vivo situation [30].

Several factors can influence T2 values in an ex vivo study. First of all, the temperature is different during the measurement. In in vivo measurements, joints have body temperature, whereas ex vivo measurements take place at room temperature. Differences in temperature can cause changes in signal intensity. In addition, quantitative ex vivo MRI experiments usually have different acquisition parameters. For example, the number and duration of echo times, acquisition matrix and field of view may vary when compared to an in vivo study [16,36]. Another relevant parameter is sample storage. Samples in an ex vivo study are usually fixed in formalin, which influences the hydration state of the tissue and can, therefore, have a considerable impact on the imaging values [16,37].

As shown in other studies and also demonstrated in our results, it can be difficult to detect differences in T2 mapping between patients with no and only mild signs of osteoarthritis [34]. However, Wang et al. found significantly higher T2 values in acutely injured stifles (in the case of a ruptured cranial cruciate ligament) compared to healthy subjects [14].

T2 mapping of the menisci can be challenging with regard to the heterogeneity of meniscal tissue [38,39,40]. In previous studies, zonal differences in quantitative MRI parameters were described, with the lowest values presented in the middle zone of the meniscus [16,33,37,39]. Even in healthy subjects, there was a certain degree of heterogeneity in T2 relaxation times [15]. In addition, due to differences in mechanical pressure and collagen content, degenerative changes vary between meniscal zones [37]. This is probably the main reason why differentiation between healthy patients and patients with only light signs of meniscal degeneration proves difficult.

Nebelung and colleagues [16] compared multiple quantitative MRI parameters, such as T1, T1p, T2, T2* and UTE T2*, with histology as a reference standard in an ex vivo study design [16]. They found a significant increase in all imaging parameters except for T2*. They also reported a significant positive and strong correlation of MRI parameters with histological scores. Eijgenraam and colleagues [30] also emphasized the power of T2 relaxation time as a non-invasive biomarker in the case of osteoarthritis. They found a stronger correlation between T2 and histologic scoring compared to the data of the present study, which may be attributed to their smaller sample size and higher osteoarthritis grade in patients. It is possible that MR imaging techniques that achieve extremely short echo times, such as UTE and T2, are more suitable for quantifying menisci matrix composition than standard spin-echo techniques that rely on T2 mapping [33,40,41]. In the aforementioned study by Nebelung et al. [16], the results of UTE T2* mapping were comparable to those of T2 mapping.

The present results show that T2 mapping should be used with caution as a biomarker for early meniscal degeneration. The results of this study suggest that there is a certain degree of histological change in menisci, even if there are no altered T2 relaxation times. Critical consideration must be given to whether the degenerative meniscal changes are of clinical relevance. The degeneration of menisci found in this study may reflect normal age-related changes because we examined menisci of elderly dogs.

T2 mapping allows for a relatively wide range of TEs. It allows both short and longer TEs. Short TEs are useful for the assessment of menisci and longer TEs may be useful to assess other joint structures, for example the articular cartilage [9,26,31,32]. T2 mapping can thus be a good tool to detect early matrix changes in joint structures. Early detection of such changes can improve the understanding of osteoarthritis development. It also helps to initiate therapy as early as possible to slow the progression of the disease. The objective is to identify patients at risk of osteoarthritis before the onset of clinical symptoms.

The limitations of the present study included the inhomogeneous study population, which was due to the fact that only cadavers from an animal hospital were accessible as study samples. The dogs varied in breed, sex, weight and age. Additionally, most of the dogs showed only slight signs of osteoarthritis. Therefore, the radiological and histological differences between the individual joints were rather slight. The results would have been statistically stronger if a group of healthy animals and a group of animals with severe degrees of osteoarthritis. The menisci showed a relatively small range in the histologic score. This was possibly due to the limited number of histologic slides for each meniscus. Furthermore, the histological scoring did not differentiate between the meniscal zones. For more information on zonal details and differences, a separate examination of the anterior horn, middle zone and posterior horn would be beneficial.

Future studies should include a larger and more homogeneous study population. In addition, meniscal zones should be subdivided according to anatomic features for both T2 mapping and histologic examination.

## 5. Conclusions

In summary, our results suggest that there are histological degenerations in menisci of older dogs, even if there are no significant radiological signs of osteoarthritis, including no significant changes in T2 relaxation time.

## Figures and Tables

**Table 1:** Information about study population.

Number of menisci	32
Number of patients	8
Median age Mean age Range (age)	13 12.94 10–17
Median body weight Mean body weight Range (body weight)	27.5 25.5 20–30
Median BCS Mean BCS Range (BCS)	6 6 4–7
Female (neutered)	4
Male	3
Female	1

**Table 2:** Statistical values of total histological score for medial and lateral menisci.

**Total score (all menisci)**	
Median Mean Range	4.5 4.25 0–7
**Total score (medial menisci)**	4.25
Median Mean Range	5 4.75 0–7
**Total score (lateral menisci)**	
Median Mean Range	4 3.75 0–6

**Table 3:** Percentage distribution of total histological score for medial and lateral menisci.

Total Score	Medial Menisci	Lateral Menisci
Score 0	6.25%	6.25%
Score 1	-	-
Score 2	-	18.75%
Score 3	6.25%	6.25%
Score 4	25%	31.25%
Score 5	31.25%	31.25%
Score 6	18.75%	6.25%
Score 7	12.5%	-
Score 8	-	-
Score 9	-	-
Score 10	-	-

**Table 4:** Percentage distribution of scoring of collagen content in medial and lateral menisci.

**Scoring of collagen content**	**Medial menisci**	**Lateral menisci**
Score 0	18.75%	25%
Score 1	56.25%	56.25%
Score 2	25%	18.75%
**Scoring of proteoglycan content**	**Medial menisci**	**Lateral menisci**
Score 0	18.75%	81.25%
Score 1	37.5%	12.5%
Score 2	43.75%	6.25%
**Scoring of cellularity**	**Medial menisci**	**Lateral menisci**
Score 0	12.5%	6.25%
Score 1	56.25%	68,75%
Score 2	31.25	25%
Score 3	-	-
**Scoring of collagen organization**	**Medial menisci**	**Lateral menisci**
Score 0	6.25%	6.25%
Score 1	75%	56.25%
Score 2	18.75%	31.25
Score 3	-	6.25%

## Data Availability

The data presented in this study are available on request from the corresponding author. The data are not publicly accessible due to data protection.

## References

[1] Pownder S.L., Hayashi K., Caserto B.G., Norman M.L., Potter H.G., Koff M.F. (2018). Magnetic Resonance Imaging T2 Values of Stifle Articular Cartilage in Normal Beagles. Vet. Comp. Orthop. Traumatol..

[2] Ding C., Cicuttini F., Jones G. (2008). How important is MRI for detecting early osteoarthritis?. Nat. Clin. Pract. Rheumatol..

[3] MacFarlane L., Yang H., Collins J., Guermazi A., Jones M., Teeple E., Xu L., Losina E., Katz J. (2016). Associations among meniscal damage, meniscal symptoms and knee pain severity. Osteoarthr. Cartil..

[4] Antony B., Driban J., Price L., Lo G., Ward R., Nevitt M., Lynch J., Eaton C., Ding C., McAlindon T. (2016). The relationship between meniscal pathology and osteoarthritis depends on the type of meniscal damage visible on magnetic resonance images: Data from the Osteoarthritis Initiative. Osteoarthr. Cartil..

[5] Hu J., Xin H., Chen Z., Zhang Q., Peng Y., Jin Z. (2019). The role of menisci in knee contact mechanics and secondary kinematics during human walking. Clin. Biomech..

[6] Fox A.J., Wanivenhaus F., Burge A.J., Warren R.F., Rodeo S.A. (2014). The human meniscus: A review of anatomy, function, injury, and advances in treatment. Clin. Anat..

[7] McDermott I. (2011). Meniscal tears, repairs and replacement: Their relevance to osteoarthritis of the knee. Br. J. Sports Med..

[8] Mosher T.J., Dardzinski B.J. (2004). Cartilage MRI T2 relaxation time mapping: Overview and applications. Semin. Musculoskelet. Radiol..

[9] Oei E.H.G., van Tiel J., Robinson W.H., Gold G.E. (2014). Quantitative Radiologic Imaging Techniques for Articular Cartilage Composition: Toward Early Diagnosis and Development of Disease-Modifying Therapeutics for Osteoarthritis. Arthritis Care Res..

[10] Arno S., Bell C.P., Xia D., Regatte R.R., Krasnokutsky S., Samuels J., Oh C., Abramson S., Walker P.S. (2016). Relationship between meniscal integrity and risk factors for cartilage degeneration. Knee.

[11] Hofmann F.C., Neumann J., Heilmeier U., Joseph G.B., Nevitt M.C., McCulloch C.E., Link T.M. (2017). Conservatively treated knee injury is associated with knee cartilage matrix degeneration measured with MRI-based T2 relaxation times: Data from the osteoarthritis initiative. Skelet. Radiol..

[12] Banjar M., Horiuchi S., Gedeon D.N., Yoshioka H. (2022). Review of Quantitative Knee Articular Cartilage MR Imaging. Magn. Reson. Med. Sci..

[13] Baum T., Joseph G., Karampinos D., Jungmann P., Link T., Bauer J. (2013). Cartilage and meniscal T2 relaxation time as non-invasive biomarker for knee osteoarthritis and cartilage repair procedures. Osteoarthr. Cartil..

[14] Wang A., Pedoia V., Su F., Abramson E., Kretzschmar M., Nardo L., Link T.M., McCulloch C.E., Jin C., Ma C.B. (2015). MR T1ρ and T2 of meniscus after acute anterior cruciate ligament injuries. Osteoarthr. Cartil..

[15] Rauscher I., Stahl R., Cheng J., Li X., Huber M.B., Luke A., Majumdar S., Link T.M. (2008). Meniscal Measurements of T1ρ and T2 at MR Imaging in Healthy Subjects and Patients with Osteoarthritis. Radiology.

[16] Nebelung S., Tingart M., Pufe T., Kuhl C., Jahr H., Truhn D. (2016). Ex vivo quantitative multiparametric MRI mapping of human meniscus degeneration. Skelet. Radiol..

[17] Diekmann H.U. Analysis of the pathogenesis and progression of osteoarthritis in canine stifle joints considering three bone healing markers. 13 September 2022. https://elib.tiho-hannover.de/receive/etd_mods_00000009.

[18] Kellgren J.H., Lawrence J.S. (1957). Radiological assessment of osteo-arthrosis. Ann. Rheum. Dis..

[19] Puchtler H., Waldrop F.S., Valentine L.S. (1973). Polarization microscopic studies of connective tissue stained with picro-sirius red FBA. Beitr Pathol..

[20] Junqueira L.C., Bignolas G., Brentani R.R. (1979). Picrosirius staining plus polarization microscopy, a specific method for collagen detection in tissue sections. Histochem. J..

[21] Whittaker P. (1995). Polarlzed light microscopy in biomedical research. Microsc. Anal..

[22] Romeis B. (1989). Mikroskopische Technik. 17. Aufl.

[23] Sun Y., Mauerhan D.R., Kneisl J.S., Norton H.J., Zinchenko N., Ingram J., Hanley E.N., Gruber H.E. (2012). Histological Exam-ination of Collagen and Proteoglycan Changes in Osteoarthritic Menisci. Open Rheumatol. J..

[24] Pauli C., Grogan S., Patil S., Otsuki S., Hasegawa A., Koziol J., Lotz M., D’Lima D. (2011). Macroscopic and histopathologic analysis of human knee menisci in aging and osteoarthritis. Osteoarthr. Cartil..

[25] Harper T.A.M., Jones J.C., Saunders G.K., Daniel G.B., Leroith T., Rossmeissl E. (2011). Sensitivity of low-field T2 images for detecting the presence and severity of histopathologic meniscal lesions in dogs. Veter. Radiol. Ultrasound.

[26] Hayashi K., Caserto B.G., Breighner R.E., Norman M.L., Potter H.G., Koff M.F., Pownder S.L. (2018). Quantitative Magnetic Resonance Imaging and Histological Comparison of Normal Canine Menisci. Vet. Comp. Orthop. Traumatol..

[27] Jackson J., Vasseur P.B., Griffey S., Walls C.M., Kass P.H. (2001). Pathologic changes in grossly normal menisci in dogs with rupture of the cranial cruciate ligament. J. Am. Veter. Med. Assoc..

[28] Roughley P.J., Lee E.R. (1994). Cartilage proteoglycans: Structure and potential functions. Microsc Res Tech..

[29] Dijkgraaf L.C., de Bont L.G., Boering G., Liem R.S. (1995). Normal cartilage structure, biochemistry, and metabolism. J. Oral Maxillofac. Surg..

[30] Eijgenraam S.M., Bovendeert F.A.T., Verschueren J., van Tiel J., Bastiaansen-Jenniskens Y.M., Wesdorp M.A., Nasserinejad K., Meuffels D.E., Guenoun J., Klein S. (2019). T2 mapping of the meniscus is a biomarker for early osteoarthritis. Eur. Radiol..

[31] Li X., Ma C.B., Link T., Castillo D.-D., Blumenkrantz G., Lozano J., Carballido-Gamio J., Ries M., Majumdar S. (2007). In vivo T1ρ and T2 mapping of articular cartilage in osteoarthritis of the knee using 3T MRI. Osteoarthr. Cartil..

[32] Li H., Chen S., Tao H., Chen S. (2015). Quantitative MRI T2 Relaxation Time Evaluation of Knee Cartilage. Am. J. Sports Med..

[33] Williams A., Qian Y., Golla S., Chu C. (2012). UTE-T2∗ mapping detects sub-clinical meniscus injury after anterior cruciate ligament tear. Osteoarthr. Cartil..

[34] Zarins Z., Bolbos R., Pialat J., Link T., Li X., Souza R., Majumdar S. (2010). Cartilage and meniscus assessment using T1rho and T2 measurements in healthy subjects and patients with osteoarthritis. Osteoarthr. Cartil..

[35] Mittal S., Pradhan G., Singh S., Batra R. (2019). T1 and T2 mapping of articular cartilage and menisci in early osteoarthritis of the knee using 3-Tesla magnetic resonance imaging. Pol. J. Radiol..

[36] Van Tiel J., Kotek G., Reijman M., Bos P.K., Bron E.E., Klein S., Nasserinejad K., Van Osch G.J.V.M., Verhaar J., Krestin G.P. (2016). Is T1ρ Mapping an Alternative to Delayed Gadolinium-enhanced MR Imaging of Cartilage in the Assessment of Sulphated Glycosaminoglycan Content in Human Osteoarthritic Knees? An in Vivo Validation Study. Radiology.

[37] Son M., Goodman S., Chen W., Hargreaves B., Gold G., Levenston M. (2013). Regional variation in T1ρ and T2 times in osteoarthritic human menisci: Correlation with mechanical properties and matrix composition. Osteoarthr. Cartil..

[38] Ghadially F.N., Lalonde J.M., Wedge J.H. (1983). Ultrastructure of normal and torn menisci of the human knee joint. J. Anat..

[39] Tsai P.-H., Chou M.-C., Lee H.-S., Lee C.-H., Chung H.-W., Chang Y.-C., Huang G.-S. (2009). MR T2 values of the knee menisci in the healthy young population: Zonal and sex differences. Osteoarthr. Cartil..

[40] McWalter E.J., Gold G.E. (2012). UTE T2∗ mapping detects sub-clinical meniscus degeneration. Osteoarthr. Cartil..

[41] Seneag D.B., Shah P., Koff M.F., Lim W.Y., Rodeo S.A., Potter H.G. (2014). Quantitative Ultrashort Echo Time Magnetic Resonance Imaging Evaluation of Postoperative Menisci: A Pilot Study. HSS J..

